# Comparison of microcapillary column length and inner diameter investigated with gradient analysis of lipids by ultrahigh‐pressure liquid chromatography‐mass spectrometry

**DOI:** 10.1002/jssc.202000545

**Published:** 2020-10-07

**Authors:** Kelsey E. Miller, James W. Jorgenson

**Affiliations:** ^1^ Center for Environmental Measurement and Modeling U.S. Environmental Protection Agency Research Triangle Park North Carolina 27709 USA; ^2^ Department of Chemistry University of North Carolina at Chapel Hill Chapel Hill North Carolina 27599 USA

**Keywords:** gradient elution, lipidomics, mass spectrometry, peak capacity, ultrahigh‐pressure liquid chromatography

## Abstract

Biological samples in lipidomic studies can consist of extremely complex mixtures due to the diverse range of species and isomerism. Herein, highly efficient, in‐house packed microcapillary columns introduce the potential to better separate these complex mixtures. We compared the effects of changing column length (15, 30, and 60 cm) and inner diameter (75 and 100 μm) on lipid separation efficiency by reversed‐phase gradient analysis using ultrahigh‐pressure liquid chromatography coupled to mass spectrometry with operating pressures ranging from 450 to 2200 bar. Seven lipid standards composed of phosphatidylcholine and triacylglycerol species were analyzed at four different gradient rates to calculate conditional peak capacity. The longest column, 60 cm, at the shallowest gradient of 2% gave the highest peak capacity of 359 with a separation window of 2 h. The intermediate column length of 30 cm with 75 μm inner diameter provided a peak capacity of 287 with a separation window of 1 h. There was no significant difference in peak capacity between 75 and 100 μm inner diameter columns. This study showed that using highly efficient microcapillary columns increased peak capacity and resolution of lipids, and thus, this technique seems promising for enhancing lipid coverage and enabling better discovery of lipid biomarkers.

**Article Related Abbreviations**
ECN: equivalent carbon number
FA: fatty acid
*h*_min_: minimum reduced plate height
PC: phosphatidylcholine
R: resolution
TG: triacylglycerol

## 1 INTRODUCTION

Lipidomics, a subset of metabolomics, can reveal changes in phenotype and cellular function by monitoring the lipid profiles within organisms [1]. Over the past two decades, the field of lipidomics has uncovered important functions and pathways of lipids, which can be used to elucidate the difference between normal and diseased states [2, 3, 4]. This research has important implications in discovering potential biomarkers in illnesses such as diabetes [5], cardiovascular disease [6], and Alzheimer's disease [7]. Continuing to better understand diseases and biological systems first requires improvements to analytical technologies and methodologies that can overcome issues innate to lipidomics.

Lipidomics research presents analytical challenges due to the immense diversity and variability among lipid classes and isomerism. The diversity in lipid structure allows these molecules to serve numerous biological functions such as provide the foundation for cellular membranes [8], act as a mechanism for energy storage [9], and contribute to cell signaling [10]. The number of lipid species found in biological systems is estimated to be on the order of hundreds of thousands [11]. This estimate is based on the fact that lipid structures can be arranged in many different ways. For example, the lipid structure can vary in the fatty acid (FA) chain length and the number and position of double bonds. Besides the sheer number of lipids that can exist in a sample, another analytical challenge is the number of isomers that can exist at one molecular mass. The different types of isomerism include *sn* isomerism, double bond position isomerism, *R*/*S* isomerism, and *E*/*Z* isomerism. Supporting Information Fig. S1 illustrates and explains these different isomers with an example lipid.

Two common analytical capabilities employed in lipidomics include shotgun techniques, such as direct infusion mass spectrometry, and separation methods like gas, liquid, or supercritical fluid chromatography [12, 13, 14, 15]. While direct infusion mass spectrometry offers high throughput, the analysis can suffer from matrix effects, ion suppression, and difficulty distinguishing between isobars and isomers. The use of front‐end separation before the mass spectrometer decreases sample complexity at each moment in time and reduces ion suppression, increases sensitivity, and adds additional qualitative information. Compared to direct infusion, online separation expands the potential of identifying more lipids.

To address the challenges of lipidomics research (e.g., isobars, isomers, low abundant species, a wide range of solute polarities), a high throughput, high sensitivity, and high resolving power technique is needed. Capillary UHPLC (typically columns with inner diameters ranging from 10 to 150 μm) meets these requirements because it is one of the most sensitive, efficient, and high resolution techniques available [16, 17]. By increasing column length or decreasing particle size, resolving power can be increased further. However, changing these parameters comes at the expense of increased backpressure. Furthermore, the viscous solvents typically needed in lipidomics studies increase the backpressure as well [18]. One solution is to increase operation pressure, but most commercial UHPLC systems can provide pressures only up to 1400 bar [19]. A modified commercial UHPLC system previously described in the literature can achieve pressures up to 2800 bar [20]. In addition, our highly efficient in‐house packed columns with sub‐2 μm particles produce about 500,000 theoretical plates per meter and achieve minimum reduced plate height (*h*_min_) values approaching 1 [21]. The high resolving power and increased peak capacity of these columns introduce the potential to better separate complex lipid mixtures. Improvement in column efficiency will allow for the introduction of more sufficiently resolved lipids into the mass spectrometer and increase lipid identifications.

Investigations into column efficiency and peak capacity have been more common in proteomics than metabolomics most likely because proteomics is a more established field [8, 22–25]. Grinias et al. separated a standard protein digest in 700 min using a 1 m column and achieved a peak capacity of 877 [20]. A study conducted by Shen et al. separated *Shewanella oneidensis* metabolomes on their 20 kpsi UHPLC system and achieved a peak capacity of 1500 using a 2‐m column and 1720 min separation window [26]. To obtain these high peak capacities in single dimension LC is a huge feat. Currently, there is no comprehensive work that investigates the effects of long capillary columns on peak capacity for lipids besides a recent article by Sorensen et al. [27]. This study compared the peak capacity of varying column lengths and columns into which silica particles were packed under different circumstances (i.e., packing while the column is in a sonication bath or not). The long columns (50 cm) that were sonicated provided the best peak capacity of 410 ± 5 (n  =  3).

Herein, we compared the column efficiency of sonicated microcapillary columns at various lengths and inner diameters (id) and demonstrated how changing these parameters and the gradient rate affect lipid separation in terms of conditional peak capacity and resolution. Columns were packed with sub‐2 μm fully porous particles and characterized by van Deemter analysis. Afterward, a gradient analysis of each column was performed by calculating conditional peak capacity of lipid standards that were separated using the modified UHPLC‐MS system. A RP gradient was used with high pressures ranging from 450 to 2200 bar at four different gradient rates.

## 2 MATERIALS AND METHODS

### 2.1 Chemicals and materials

HPLC‐grade acetonitrile (ACN), acetone, hexane, methanol, dichloromethane, tetrahydrofuran, trifluoroacetic acid, l‐ascorbic acid, hydroquinone, resorcinol, catechol, 4‐methylcatechol and LC‐MS grade water, water with 0.1% formic acid (v/v), ACN, ACN with 0.1% formic acid, isopropanol (IPA), formic acid, and ammonium formate were purchased from Fisher Scientific (Fair Lawn, NJ). Deionized water was collected from a Nanopure ultrapure water system (Barnstead International, Dubuque, IA). For fabrication of kasil frits, potassium silicate was purchased from PQ Corporation (Valley Forge, PA), and formamide and Whatman 934‐AH 125 mm glass microfiber filter paper (GE Healthcare, UK) were purchased from Sigma‐Aldrich (St. Louis, MO). The phosphatidylcholine (PC) standards: PC (14:0/14:0), PC (16:1/16:1), PC (18:1/18:1)‐ω9‐*Z*, PC (18:1/18:1)‐ ω9‐*E*, and PC (18:0/18:2) were purchased from Avanti Polar Lipids (Alabaster, AL). The triacylglycerol (TG) standards TG (18:1/18:1/18:1) and TG (18:0/18:0/18:0) were purchased from Sigma‐Aldrich (St. Louis, MO).

Cylindrical fused‐silica tubing with 72.9 μm id and 361.5 μm outer diameter (od), 100.5 μm id and 357.5 μm od, and 20.3 μm id and 360.7 μm od were obtained from Polymicro Technologies (Phoenix, AZ). The gradient storage loop was 50 m long with 76.2 μm id and 357.6 μm od cylindrical fused‐silica tubing. Waters Corporation (Milford, MA) provided C18‐modified 1.7 μm BEH silica particles. These particles had a Sauter diameter of 1.92 μm, which was calculated from a SEM based on particle size distribution obtained from the measurement of approximately 869 BEH particles from the same batch using a JSM‐7500F SEM (JEOL, München, Germany). The PicoClear connector used to connect the column outlet to the empty capillary tubing (20 cm × 20 μm id × 360 μm od) and the 20 μm × 360 μm × 6.35 cm pulled tip (10 μm) uncoated nanospray SilicaTip emitters were purchased from New objective (Woburn, MA).

### 2.2 Preparation of capillary ultrahigh‐pressure LC columns

Details of the column packing procedure can be found in an article by Godinho et al. [21]. Briefly, capillary columns were packed with a high slurry concentration of 200 mg/mL while ultrasonication was applied. The only difference in procedure was that different lengths and inner diameters of empty capillary tubing were packed. Additionally, a different (compared to [21]) batch of BEH C18 particles and capillary were used.

Three columns were prepared as column replicates in four sets for a total of 12 columns. Three sets of columns, with lengths of 15, 30, and 60 cm and id of 75 μm, were evaluated. Additionally, another set of columns with three column replicates, which had dimensions of 30 cm × 100 μm id, was evaluated.

### 2.3 van Deemter characterization of columns

Electrochemical detection was performed isocratically with a mobile phase of 50/50 water/ACN + 0.1% trifluoroacetic acid (v/v/v). Small molecule analytes included hydroquinone, resorcinol, catechol, and 4‐methylcatechol with ascorbic acid as the dead time marker. This electrochemical detection has been described in more detail previously [21]. An algorithm written in Igor Pro 6.0 (Wavemetrics, Lake Oswego, OR) used the reduced plate height (h) and reduced velocity (v) data collected from the van Deemter analysis to apply a best fit van Deemter curve for each standard. Using the van Deemter equation, the best fit provided the reduced *a*, *b*, and *c* coefficients, which were then used to calculate each standard's *h*_min_.

Since van Deemter analyses were performed with ∼100 Da standards, we estimated that the van Deemter optimal velocity was slower for lipids with molecular weights between 700 and 1000 Da moving through the significantly more viscous mobile phases encountered in the gradient analyses. van Deemter analyses were not performed with lipid standards, so we performed hypothetical calculations to estimate what the optimal interstitial velocity would be for each column id (75 and 100 μm), and these calculations are explained in the Supporting Information. These optimal velocities were compared to the calculated experimental velocities used during the gradient analyses. The viscous mobile phase conditions, a lipid's molecular weight, and other experimental factors were taken into account to estimate a lipid's diffusion coefficient. Briefly, the cross‐sectional area of the column was increased by about two when using 100 μm id column instead of 75 μm id, resulting in a calculated experimental interstitial velocity that was roughly halved at the same flow rate of 300 nL/min (0.14 vs. 0.25 cm/s). Decreasing the interstitial velocity while maintaining the same volumetric flow rate allowed the 30 cm × 100 μm columns to perform closer to the calculated van Deemter optimal velocity (0.036 cm/s) than 30 cm × 75 μm columns (0.034 cm/s). Although both column ids had experimental interstitial velocities higher than the optimal velocity, we investigated whether increasing the column id to 100 μm could result in a higher peak capacity compared to 30 cm × 75 μm columns that operated at a faster interstitial velocity.

### 2.4 Sample preparation for ultrahigh‐pressure LC‐MS analysis

Stock solutions were prepared by dissolving PC standards in dichloromethane/methanol (2:1 v/v). TG (18:1/18:1/18:1) standard was dissolved in hexane/IPA (1:1 v/v) and TG (18:0/18:0/18:0) standard was dissolved in tetrahydrofuran. PC sample concentration ranged from 0.015 to 1.25 μg/mL diluted in mobile phase A solution. TG sample concentration ranged from 0.015 to 6 μg/mL diluted in mobile phase B solution. All PC standards were in one sample vial while all TG standards were in a separate sample vial due to solubility issues.

### 2.5 Chromatographic and mass spectrometric conditions

Analyses were performed on a Waters nanoACQUITY UPLC® system interfaced to a Waters Xevo QTOF MS® and the system was modified to allow for high pressures through a connection of empty capillary tubing, valves, and microvolume connector‐tees purchased from Valco Instrument (Houston, TX). The commercial UHPLC was used for loading the gradient and sample into the gradient storage loop. Specifics of the instrumental design have been discussed in detail previously [20]. The only difference between the current study and [20] is that the current study used a 75 μm id gradient storage loop. A Haskel pneumatic amplifier pump (DSXHF‐903) was used as the high pressure pump (Burbank, CA). The following pressures were used for each set of columns: 450 bar (15 cm × 75 μm), 1080 bar (30 cm × 75 μm), 2200 bar (60 cm × 75 μm), and 620 bar (30 cm × 100 μm). The combination of pressure chosen for each column dimension and an operating temperature of 60°C allowed for an approximate flow rate of 300 nL/min. This flow rate was chosen because it provided a stable spray for the nanoelectrospray ionization source and it fell within the suggested flow rate range given for the nanospray emitter.

RP conditions were used. Mobile phase A was 60% ACN and 40% water modified with 10 mM ammonium formate and 0.1% formic acid (v/v/v). Mobile phase B was 100% IPA modified with 10 mM ammonium formate and 0.1% formic acid (v/v/v). The binary gradient started at 0% mobile phase B and increased to 99% during a specified time depending on whether a 2, 4, 8, or 16% gradient rate was used. Gradient rates were determined using column volumes of 0.0297 μL/cm for 75 μm id columns and 0.0528 μL/cm for 100 μm id columns [28]. It is important to note that due to the modified UHPLC's use of a constant pressure pump instead of a constant flow pump, as is used in commercial LCs, gradient rates used in this article are based on volume and are % change of mobile phase B composition per column volume (%Δ/cv). A more detailed explanation of this equation can be found in the Supporting Information.

A double injection was performed with the first injection sampling 0.2 μL from the PC vial and the second injection sampling 1 μL from the TG vial. A 3.5 μL plug of mobile phase A was inserted between the two samples and also after the TG sample. Positive ion mode was used under the following conditions: capillary voltage, 2.0 kV; cone voltage, 30 V; source temperature, 70°C; collision gas, argon; cone gas (N_2_), 40 L/h; nanoflow gas (N_2_), 0.30 bar; purge gas (N_2_), 250 L/h. Data were collected using MassLynx V4.1 SCN833 in MS^E^ acquisition mode between m/z 615 and 1000 Da with a scan duration of 0.6 s.

### 2.6 Conditional peak capacity characterization

One way to describe the quality of a separation by gradient elution is to use peak capacity. In this study, we use what is known as conditional peak capacity, which is described as the number of peaks that can be resolved (4σ) in a defined separation window [29]. A high peak capacity indicates that components in a complex mixture are better resolved in a separation window. The same suggestions for improving resolution apply to increasing peak capacity (e.g., increasing column length, decreasing particle size, and improving column packing procedures). Furthermore, peak capacity is inversely proportional to gradient rate and eventually peak capacity will plateau as the gradient rate becomes increasingly shallow.

After each UHPLC‐MS analysis, the chromatogram list was imported into Igor Pro 6.0. An algorithm written in Igor performed a Gaussian fit onto each of the seven peaks, which then provided the 4σ peak width (13.4% of the maximum peak height) and retention time. The separation window was calculated by subtracting the retention time of the first eluting compound PC (14:0/14:0) from the last eluting compound TG (18:0/18:0/18:0). The arithmetic mean of all seven peak widths was calculated. The equations used to calculate conditional peak capacity and resolution can be found in the Supporting Information [29, 30].

## 3 RESULTS AND DISCUSSION

### 3.1 van Deemter characterization of capillary columns

The effects of column length and capillary id on the efficiency of separation were studied with low molecular weight standards and electrochemical detection. A summary of each column's separation efficiency can be found in Table 1. Supporting Information Fig. S2 illustrates van Deemter curves for a representative column from each set. All van Deemter curves are flat across a wide range of velocities indicating that the c‐term was low. Supporting Information Table S1 details the reduced van Deemter coefficients and the *h*_min_ values of hydroquinone (*k*′ = 0.2) for each column. Maximum theoretical plate numbers (*N*_max_) were rounded to two significant figures after calculations that used 1.92 μm particle diameter and each column's respective hydroquinone *h*_min_ and column length. All columns showed similar *h*_min_ values and plate numbers grew in proportion with increasing column length as expected.

**TABLE 1:** A summary of identifying information, length, id, reduced plate height, plate number, and peak capacity (at 2% gradient rate) for each column

Color	Shape	Identifier	Length (cm)	id (μm)	*h*_min_ (HQ)	*N*_max_	Peak capacity (2%)
Black	●	15_75_1	14.6	75	1.49	51 000	187
	▲	15_75_2	15.3	75	1.52	52 000	193
	■	15_75_3	15.1	75	1.55	51 000	187
Blue	●	30_75_1	30.0	75	1.40	110 000	267
	▲	30_75_2	30.3	75	1.48	110 000	251
	■	30_75_3	30.2	75	1.32	120 000	287
Red	●	60_75_1	59.0	75	1.37	220 000	321
	▲	60_75_2	60.2	75	1.40	220 000	356
	■	60_75_3	59.8	75	1.33	230 000	359
Light blue	●	30_100_1	30.3	100	1.37	120 000	282
	▲	30_100_2	29.8	100	1.52	100 000	286
	■	30_100_3	29.8	100	1.36	110 000	263

### 3.2 Gradient analysis by ultrahigh‐pressure LC‐MS

Seven lipid standards were analyzed on the modified UHPLC‐MS to assess the quality of the gradient separation as the column length and capillary id changed. Lipid standards were selected with the purpose of having pairs that are hard to resolve, which meant choosing standards with the same equivalent carbon number (ECN) or isomers. ECN is a measure of the FA chain's polarity [31]. ECN is calculated by the total number of FA carbons minus twice the number of double bonds on the FA chains. Resolving lipids with the same ECN can be complicated because they typically have similar retention times. The standards PC (14:0/14:0) and PC (16:1/16:1), for example, possess the same ECN of 28. Standards PC (*Z* 18:1/18:1), PC (18:0/18:2), and PC (*E* 18:1/18:1) are isomers and all have an ECN of 32. Additionally, PCs were chosen as test analytes because they ionize easily in positive ion mode and showcase a range of FA chains [32]. The TG standards do not possess the same ECN, but they represent another lipid class that expands the separation window.

**FIGURE 1:**
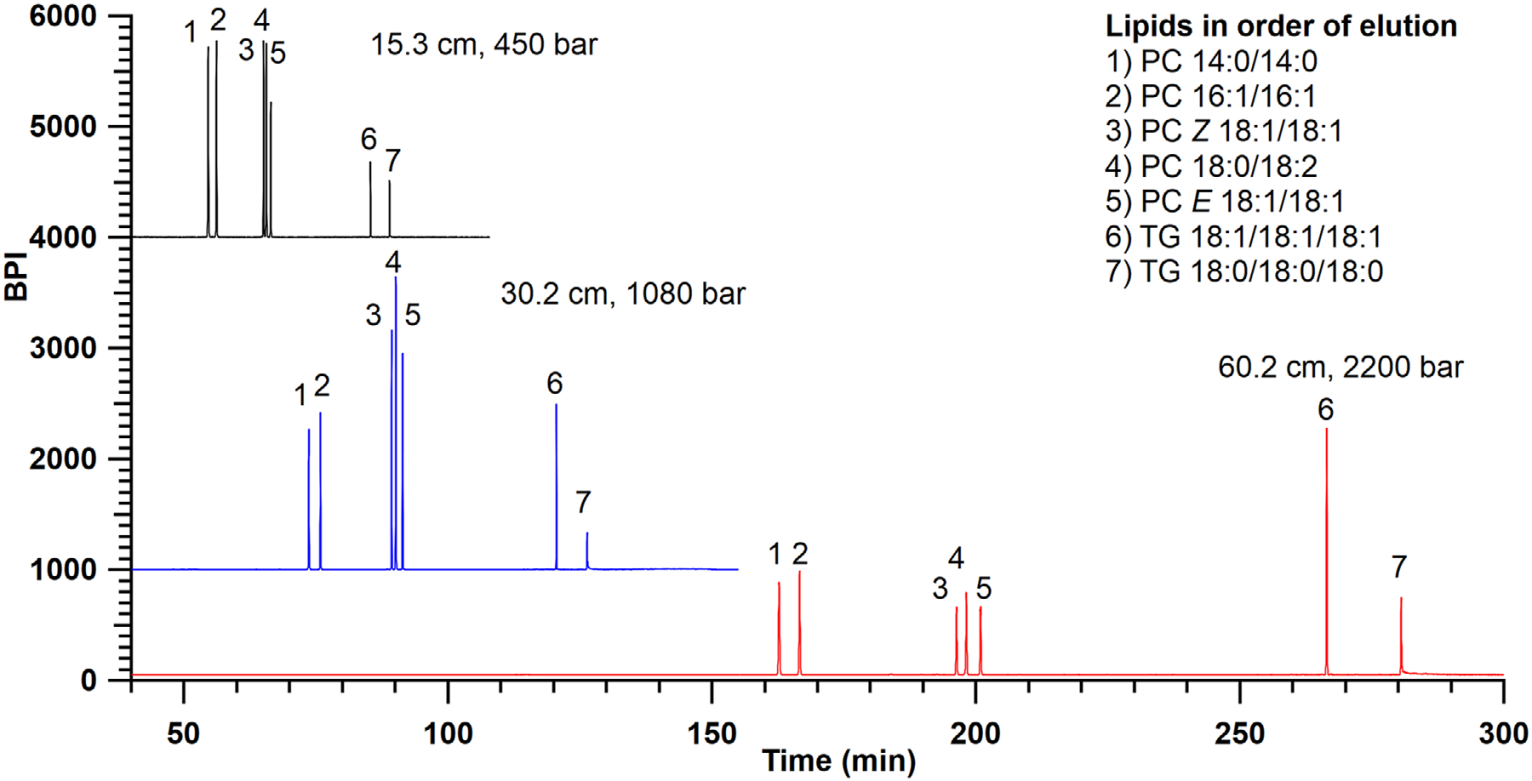
Base peak intensity (BPI) chromatograms of seven lipid standards detected in positive ion mode. Standards were separated with a 2% gradient rate on 15.3 cm × 75 μm id column (black), 30.2 cm × 75 μm id column (blue), and 60.2 cm × 75 μm id column (red)

**FIGURE 2:**
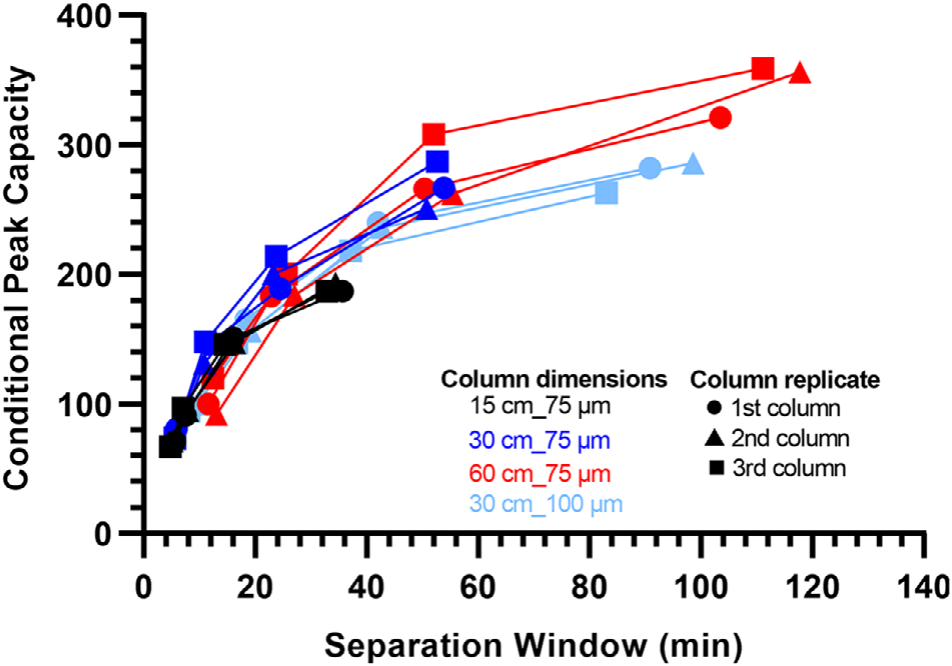
Conditional peak capacity for each column at four gradient rates (2, 4, 8, and 16%). Three column replicates (circle, triangle, square) were analyzed for each set of columns (black, blue, red, light blue)

Examples of chromatograms for 15, 30, and 60 cm columns with 75 μm id are shown in Fig. 1. The chromatograms demonstrate how the separation window increased with increasing column length. By changing the column lengths, the analysis times were fastest with 15 cm columns, intermediate with 30 cm columns, and slowest with 60 cm columns. Conditional peak capacity calculations at each gradient rate allowed for comparison among all the columns. Figure 2 illustrates that conditional peak capacity increased with longer separation windows. Four times the length from 15 to 60 cm yielded roughly twice the peak capacity. Supporting Information Table S2 provides the conditional peak capacity numbers for each column at each gradient rate. At 2% gradient, the shallowest gradient and the longest separation window, peak capacity started to plateau. The 60 cm columns provided the highest peak capacity (arithmetic mean of 345 at 2% gradient rate), but at the expense of about a 2‐h separation window, which excludes time for re‐equilibration and the beginning of the gradient. The 15 cm columns provided the lowest peak capacity (mean 189 at 2%) and the shortest analysis time. 30 cm × 75 μm columns at 2% gradient and 60 cm columns at 4% gradient gave comparable peak capacities (mean 268 vs. 278, respectively).

We compared the separation efficiency of a larger narrow‐bore column (100 μm) to 75 μm id columns, to test whether conditional peak capacity improved if a column operated closer to its optimal interstitial velocity. Since 30 cm columns provided intermediate results compared to 15 and 60 cm columns, we moved forward with 30 cm × 100 μm columns in this study. At 2% gradient, 30 cm × 100 μm columns provided no significant improvement in peak capacity over 30 cm × 75 μm columns (mean of 277 vs. 268, respectively), but required almost twice the analysis time. Therefore, moving from a faster interstitial velocity to a slower interstitial velocity does not greatly impact peak capacity in our application.

### 3.3 Comparison of column efficiency, conditional peak capacity, and resolution

**FIGURE 3:**
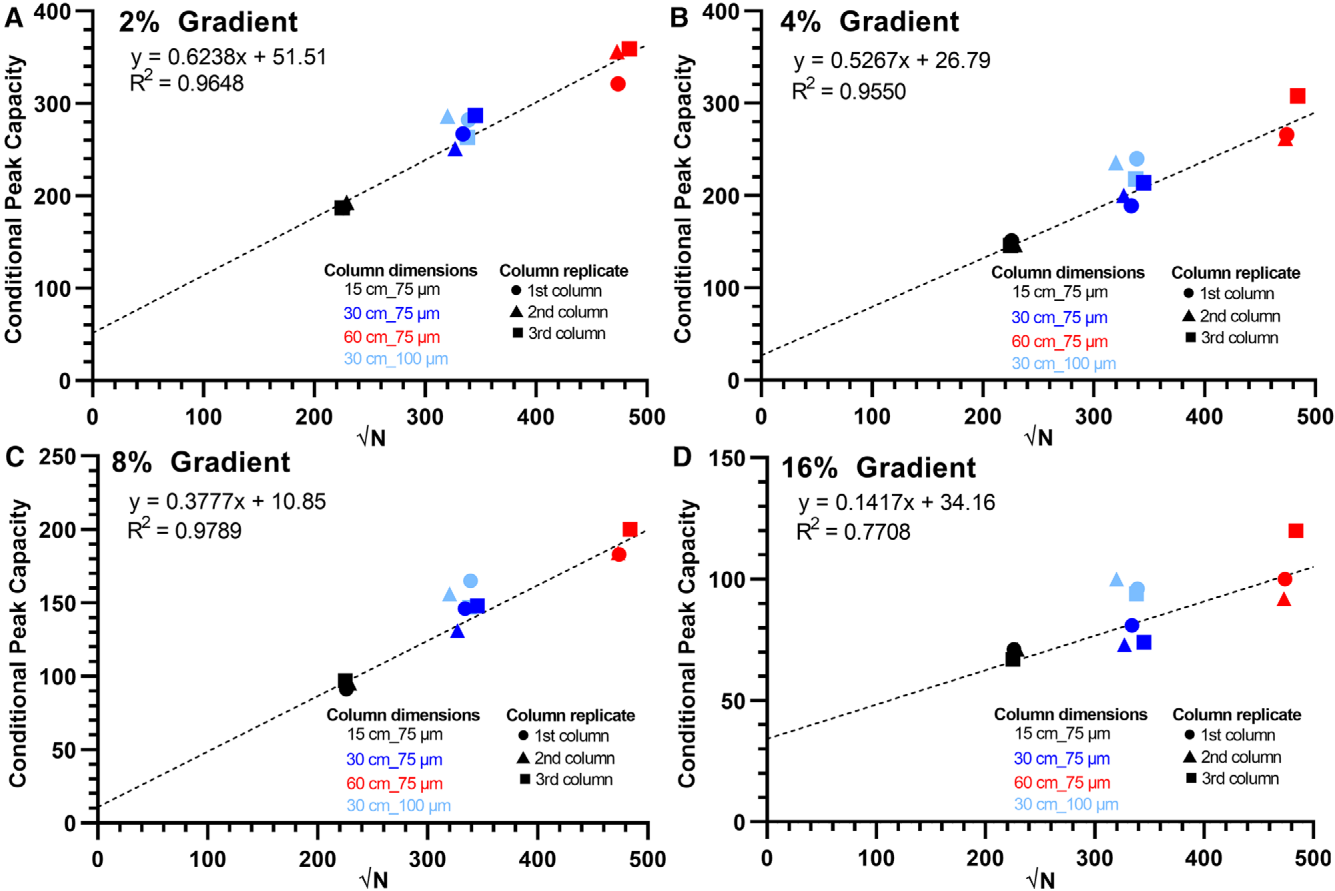
Linearity of conditional peak capacity plotted against the square root of N. Three column replicates (circle, triangle, square) were analyzed for each set of columns (black, blue, red, light blue). The linear trend calculated does not include 30 cm × 100 μm id columns. (A) 2% gradient; (B) 4% gradient; (C) 8% gradient; (D) 16% gradient

To understand the correlation between column efficiency and peak capacity, conditional peak capacity was plotted against the square root of *N* in Fig. 3. The least squares fit was applied to the data, and the linear trends showed good agreement among the three sets of 75 μm id columns that were analyzed with 2% gradient rate (*R* ^2^ value 0.9648), 4% (*R* ^2^ value 0.9550), and 8% (*R* ^2^ value 0.9789). The linear trend using 16% gradient rate was poor (*R* ^2^ value 0.7708) likely due to 16% being an extremely aggressive gradient rate. The tightest cluster among the four gradient rates was 15 cm columns while the other sets of columns showed more variance.

**FIGURE 4:**
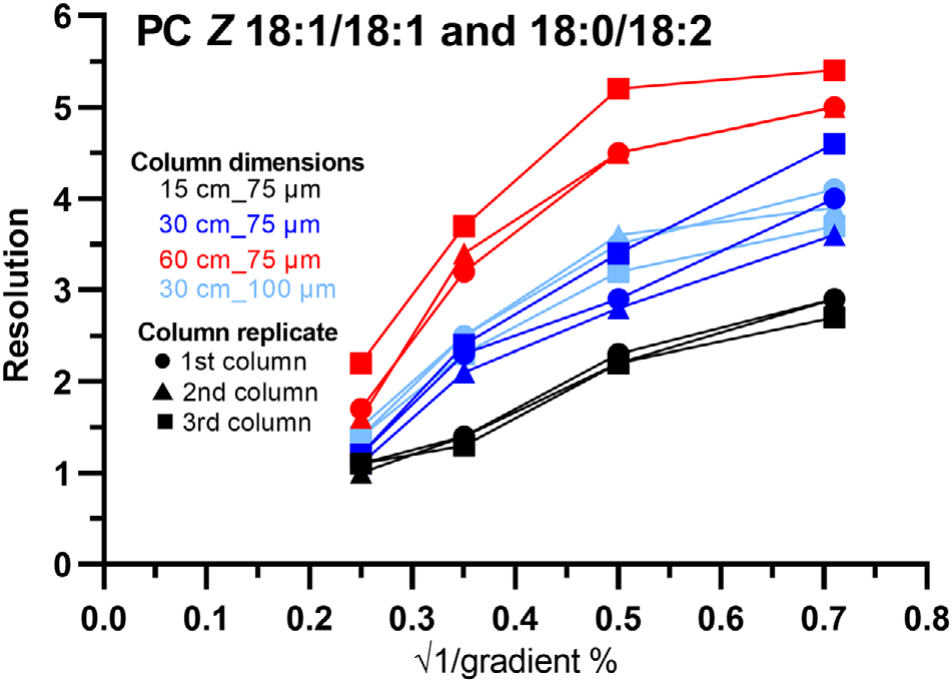
Resolution of lipid pair PC (*Z* 18:1/18:1) and PC (18:0/18:2) is plotted against the square root of the reciprocal of each gradient rate. Three column replicates (circle, triangle, square) were analyzed for each set of columns (black, blue, red, light blue)

Resolution is inversely proportional to the gradient rate and Fig. 4 depicts this relationship for lipid pair PC (*Z* 18:1/18:1) and PC (18:0/18:2) separated using the four different gradient rates [33]. To help visualize the data as a linear trend, the square root of the reciprocal of the gradient rate was plotted. Supporting Information Tables S3‐S7 list the resolution values of varying lipid pairs for each column and gradient rate and Supporting information Fig. S3 graphs resolution against the gradient rates for other lipid pairs. The critical pair, PC (*Z* 18:1/18:1) and PC (18:0/18:2), were isomers and the closest eluting pair of standards (i.e., the hardest pair to resolve). At 16% gradient rate, only 60 cm × 75 μm columns gave resolution greater than 1.5 (baseline resolution) for this critical pair. Additionally, 60 cm × 75 μm columns started to plateau with more shallow gradients. Columns (15 cm × 75 μm) achieved baseline resolution of this critical pair with a 4% gradient rate. Resolution of the lipid pair, PC (14:0/14:0) and PC (16:1/16:1), was the only pair that does not show this plateau trend with 60 cm columns. Overall, 60 cm × 75 μm columns provided the best resolution at each gradient rate and both sets of 30 cm columns gave comparable resolutions. The 15 cm columns had the lowest resolution, but at a 16% gradient rate 15 cm columns had similar resolutions to 30 cm columns.

## 4 CONCLUDING REMARKS

The effects of column length and microcapillary id on the separation of lipid standards have been studied by gradient analysis. Conditional peak capacity and resolution were measured to determine the quality of separation. Four sets of highly efficient columns packed with sub‐2 μm particles were compared. Increasing the capillary id from 75 to 100 μm did not significantly increase peak capacity but did increase the analysis time by almost a factor of two. For very complex lipid samples, the use of 60 cm × 75 μm columns using a 2% gradient rate can offer high peak capacity (mean 345), but at the expense of longer analysis time and higher pressure. Compared to 15 and 60 cm columns, 30 cm × 75 μm columns offer a compromised approach with a separation window close to 1 h that reached a mean peak capacity of about 268 using a 2% gradient rate. At 1000 bar of operating pressure, these columns easily could be run directly from a commercial system that can provide pressures up to 1500 bar. The results demonstrate that the high resolving power and increased peak capacity of these columns better separate lipids with similar ECNs or isomers. By using highly efficient capillary columns on a modified UHPLC‐MS, an analytical method exists that provides high resolution and improved separation that can be applied to analyzing complex lipid samples.

## CONFLICT OF INTEREST

The authors have declared no conflict of interest.

## Supporting information

SUPPORTING INFORMATION
