# Supplementary material for: Comparison of microcapillary column length and inner diameter investigated with gradient analysis of lipids by ultrahigh‐pressure liquid chromatography‐mass spectrometry
Source: J Sep Sci. 2020 Oct 7;43(22):4094–102. doi: 10.1002/jssc.202000545 (PMC7727313; doi:10.1002/jssc.202000545)
Supplement: Supplementary file 1 — SUPPORTING INFORMATION [file JSSC-43-4094-s001.docx]

**Supporting Information**

**S****2.3 van Deemter characterization of columns**

This section provides an explanation of hypothetical calculations that estimate what the optimal interstitial velocity of a lipid analysis would be for each column id and compares these values to the calculated experimental velocities. A lipid’s volume, the viscous mobile phase conditions, and other experimental factors were taken into account to estimate a lipid’s diffusion coefficient and the velocities.

$$V= \frac{4\pi r^{3}}{3}$$

First, volume (V) was found by multiplying the molecular mass of the solute (a lipid) by its partial specific volume. We estimated the average lipid to have a molecular weight of 800 g/mol and a partial specific volume of 1.0 cm^3^/g [1]. After rearranging the volume of a sphere equation, we used the calculated volume to solve for the solute’s radius (r). The estimated radius of a lipid was 6.82 x 10^-8^ cm.

$$f= 6\pi\eta aN$$

Second, Stokes equation was used for calculating the frictional coefficient. The solute’s radius (r) is also known as the Stokes radius (a). N is Avogadro constant (6.022 x 10^23^ mol^-1^). The viscosity (η) of the mobile phase assumed mobile phase conditions consisting of 50/50 water/2-propanol (the two most viscous components of the mobile phases used in this study). No literature could be found, in which viscosity values were measured for a ternary mixture of water, acetonitrile, and 2-propanol. Additionally, since high pressures were used in this study, it must be taken into account that this solvent mixture became more viscous at higher pressures. A study conducted by Tanaka, et al. investigated viscosity of aqueous alcohol mixtures under high pressure and differing temperatures, and those values were used in these calculations [2]. Note: the highest temperature measured in this referenced study was 323.15 K (50 °C). Given that our study incorporated a higher temperature (60 °C) and the mobile phase consisted of acetonitrile, the actual viscosity values were most likely slightly lower than what were used in these hypothetical calculations. 30 cm x 100 μm columns operated at a pressure of 620 bar, and the average pressure that the solute experienced was half of 620 bar. 310 bar closely corresponded to the viscosity value reported at a pressure of 29.5 MPa in the referenced study. A viscosity value of 1.493 mPa·s (0.01493 poise) was used for 30 cm x 100 μm columns. 30 cm x 75 μm columns operated at a pressure of 1080 bar. 540 bar closely corresponded to a viscosity value reported at a pressure of 49.1 MPa in the referenced study. A viscosity value of 1.675 mPa·s (0.01675 poise) was used for 30 cm x 75 μm columns. The frictional coefficient calculated for a lipid analyzed with a 30 cm x 100 μm column yielded 1.16 x 10^16^ g/s·mol. The frictional coefficient calculated for a lipid analyzed with a 30 cm x 75 μm column yielded 1.30 x 10^16^ g/s·mol.

$$D= \frac{RT}{f}$$

Third, the diffusion coefficient of the lipid was solved for by using R constant (8.31 J/mol·K or 8.31 x 10^7^ g·cm^2^/s^2^·mol·K), temperature (T), and the calculated frictional coefficient (f). The column heater operated at a temperature of 60 °C. The diffusion coefficient calculated for 30 cm x 100 μm columns was 2.40 x 10^-6^ cm^2^/s. The diffusion coefficient calculated for 30 cm x 75 μm columns was 2.14 x 10^-6^ cm^2^/s.

$$u= \frac{F}{\varepsilon_{i}\pi r^{2}}$$

$$v= \frac{ud_{p}}{D}$$

Finally, interstitial velocity (u) and reduced velocity (v) can be determined. Interstitial velocity can be found by dividing the flow rate (F) by the column’s cross-sectional area where r is the capillary column’s radius and interparticle porosity (ε_i_). An interparticle porosity of 0.45 was used based on previous research conducted in our laboratory [3] and a study published by Reising, et al. [4].

The experimental velocities were calculated. An experimental flow rate of 300 nL/min was used in this study. The experimental velocity for a 30 cm x 100 μm column was calculated to be 0.14 cm/s and the velocity for a 30 cm x 75 μm column was determined to be 0.25 cm/s. Reduced velocity was calculated using a particle diameter (d_p_) of 1.92 μm and the calculated diffusion coefficient respective to each column id. The reduced velocity for a 30 cm x 100 μm column was calculated to be 11.34. Reduced velocity for a 30 cm x 75 μm column was calculated to be 22.62. Using the experimental van Deemter coefficients found in this study and the calculated reduced velocity, reduced plate height (h) can be calculated for each column. Referring to Table S1 and using the van Deemter terms from column 30_100_3 (a = 0.26, b = 1.58, and c = 0.19), the reduced plate height was 2.55. Using the van Deemter terms from column 30_75_3 (a = 0.28, b = 1.60, and c = 0.17), the reduced plate height was 4.20.

The optimal interstitial velocity of a lipid analysis for each column id can be determined by converting the experimentally determined reduced velocity minimum (found from test analyte hydroquinone) to interstitial velocity using the calculated diffusion coefficients from above. These calculations assumed the same van Deemter coefficients and reduced plate height minimum would be achieved with a van Deemter analysis involving a lipid test analyte. Referring to Table S1, the experimentally determined reduced velocity minimum and reduced plate height minimum for column 30_100_3 using the test analyte hydroquinone was 2.88 and 1.36, respectively. After converting to interstitial velocity with the lipid diffusion coefficient, the optimal velocity for a lipid analysis with a 30 cm x 100 μm column would be 0.036 cm/s. This yielded an optimal flow rate of 76 nL/min. Referring to Table S1, the experimentally determined reduced velocity minimum and reduced plate height minimum for column 30_75_3 using the test analyte hydroquinone was 3.07 and 1.32, respectively. The optimal velocity for a lipid analysis with a 30 cm x 75 μm column would be 0.034 cm/s and optimal flow rate would be 41 nL/min.

Upon comparison of the calculated reduced velocities and plate heights assuming conditions specific to the experiment conducted in this study and what the optimal velocities and flow rates should be in order to achieve the reduced plate height minimum, one can see there is a vast difference. Ideally, experimental conditions operate close to the optimal velocity, yet the lipid analyses operated at experimental velocities much higher than the optimal velocity. While both column ids experienced velocities higher than the optimal velocities, 30 cm x 100 μm columns operated closer to the optimal velocity (0.14 cm/s vs. 0.036 cm/s) than 30 cm x 75 μm columns (0.25 cm/s vs. 0.034 cm/s). However, our results in the gradient elution study showed that peak capacities were essentially the same between the two types of columns.

**S2.5 Chromatographic and mass spectrometric conditions**

1. Calculating gradient rate

$$\frac{\%\Delta}{cv}= \frac{total cv x \%\Delta mobile phase B}{total gradient volume}$$

The gradient rate is the rate of change of mobile phase B composition per column volume (%Δ/cv). Total column volume is determined by multiplying the column volume (μL/cm) by the column’s length (cm). %Δmobile phase B is the total percent change of mobile phase B throughout the gradient (%B_final_-%B_initial_), which is 99% in this study. The total gradient volume is the total volume used during the entire gradient. Since we knew what gradient rates we wanted to use, we rearranged the gradient rate equation to solve for the total gradient volume. The total gradient volume is calculated from the time it took to load the gradient multiplied by the flow rate, which is programmed through Waters software. Gradients were preloaded into the gradient storage loop by the commercial UPLC.

**S2.6 Conditional peak capacity characterization**

1. Calculating conditional peak capacity

$$n_{c}= \frac{gradient time (t_{g})}{peak width (4\sigma)}+1$$

Conditional peak capacity (n_c_) is the number of peaks that can be resolved ($4\sigma)$ in a defined separation window. Gradient time (t_g_) is the separation window or the time from the first eluting peak to the last eluting peak and peak width is the width at 4σ. For a Gaussian peak the 4σ peak width is the width of the peak at 13.4% of the maximum peak height.

1. Calculating resolution (R)

$$R= \frac{2(t_{R2}- t_{R1})}{w_{1}+ w_{2}}$$

t_R2_ is the retention time of the second peak being measured, t_R1_ is the retention time of the first peak being measured, w_2_ is the 4σ peak width of the second peak being measured, and w_1_ is the 4σ peak width of the first peak being measured.

| Color | Shape | Identifier | a | b | c | Best fit calculated h_min_ (HQ) | Best fit calculated v_min_ (HQ) |  |
| --- | --- | --- | --- | --- | --- | --- | --- | --- |
| Black | ● | 15_75_1 | 0.30 ± 0.13 | 1.70 ± 0.074 | 0.21 ± 0.028 | 1.49 | 2.85 |  |
|  | ▲ | 15_75_2 | 0.53 ± 0.13 | 1.54 ± 0.088 | 0.16 ± 0.027 | 1.52 | 3.10 |  |
|  | ■ | 15_75_3 | 0.45 ± 0.077 | 1.58 ± 0.058 | 0.19 ± 0.015 | 1.55 | 2.88 |  |
| Blue | ● | 30_75_1 | 0.33 ± 0.12 | 1.60 ± 0.087 | 0.18 ± 0.022 | 1.40 | 2.98 |  |
|  | ▲ | 30_75_2 | 0.031 ± 0.068* | 2.51 ± 0.067 | 0.21 ± 0.012 | 1.48 | 3.46 |  |
|  | ■ | 30_75_3 | 0.28 ± 0.071 | 1.60 ± 0.061 | 0.17 ± 0.013 | 1.32 | 3.07 |  |
| Red | ● | 60_75_1 | 0.48 ± 0.040 | 1.53 ± 0.036 | 0.13 ± 0.0071 | 1.37 | 3.43 |  |
|  | ▲ | 60_75_2 | 0.38 ± 0.023 | 1.52 ± 0.021 | 0.17 ± 0.0043 | 1.40 | 2.99 |  |
|  | ■ | 60_75_3 | 0.38 ± 0.071 | 1.52 ± 0.069 | 0.15 ± 0.013 | 1.33 | 3.18 |  |
| Light blue | ● | 30_100_1 | 0.38 ± 0.030 | 1.53 ± 0.027 | 0.16 ± 0.0052 | 1.37 | 3.09 |  |
|  | ▲ | 30_100_2 | 0.69 ± 0.30 | 1.32 ± 0.25 | 0.13 ± 0.059 | 1.52 | 3.19 |  |
|  | ■ | 30_100_3 | 0.26 ± 0.14 | 1.58 ± 0.12 | 0.19 ± 0.026 | 1.36 | 2.88 |  |

**Table S1.** Reduced van Deemter terms for each column as determined by the best fit of the van Deemter equation. Test analyte is hydroquinone (HQ) in all cases. Capacity factor (k’) of HQ is 0.2.

*The a term for this column is much smaller compared to the other columns most likely due to the need for a lower velocity sampling point. The velocity range for the characterization of this column was 0.05-0.27 cm/s.

**Table S2.** Peak capacity of each column at 2%, 4%, 8%, and 16% gradient rates.

|  |  |  | Gradient Rate | | | |
| --- | --- | --- | --- | --- | --- | --- |
|  |  |  | 16% | 8% | 4% | 2% |
| Color | Shape | Identifier | Peak Capacity | | | |
| Black | ● | 15_75_1 | 71 | 91 | 151 | 187 |
|  | ▲ | 15_75_2 | 71 | 95 | 147 | 193 |
|  | ■ | 15_75_3 | 67 | 97 | 146 | 187 |
| Blue | ● | 30_75_1 | 81 | 146 | 189 | 267 |
|  | ▲ | 30_75_2 | 73 | 131 | 200 | 251 |
|  | ■ | 30_75_3 | 74 | 148 | 214 | 287 |
| Red | ● | 60_75_1 | 100 | 183 | 266 | 321 |
|  | ▲ | 60_75_2 | 92 | 184 | 262 | 356 |
|  | ■ | 60_75_3 | 120 | 200 | 308 | 359 |
| Light blue | ● | 30_100_1 | 96 | 165 | 240 | 282 |
|  | ▲ | 30_100_2 | 100 | 156 | 236 | 286 |
|  | ■ | 30_100_3 | 94 | 147 | 218 | 263 |

**Table S3.** Resolution of PC (14:0/14:0) and PC (16:1/16:1) at gradient rates 16%, 8%, 4%, and 2%.

|  |  |  | Gradient Rate | | | |
| --- | --- | --- | --- | --- | --- | --- |
|  |  |  | 16% | 8% | 4% | 2% |
| Color | Shape | Identifier | Resolution | | | |
| Black | ● | 15_75_1 | 1.3 | 2.6 | 4.8 | 6.3 |
|  | ▲ | 15_75_2 | 1.3 | 2.6 | 4.9 | 6.7 |
|  | ■ | 15_75_3 | 1.2 | 2.7 | 4.7 | 7.3 |
| Blue | ● | 30_75_1 | 1.5 | 3.6 | 5.6 | 9.2 |
|  | ▲ | 30_75_2 | 1.5 | 3.4 | 6.5 | 8.8 |
|  | ■ | 30_75_3 | 1.8 | 4.1 | 6.6 | 10.5 |
| Red | ● | 60_75_1 | 1.5 | 3.7 | 6.7 | 9.6 |
|  | ▲ | 60_75_2 | 1.3 | 3.9 | 6.7 | 10.2 |
|  | ■ | 60_75_3 | 1.8 | 4.4 | 7.5 | 11.1 |
| Light blue | ● | 30_100_1 | 2.3 | 5.1 | 7.8 | 10.4 |
|  | ▲ | 30_100_2 | 2.4 | 4.8 | 7.9 | 10.7 |
|  | ■ | 30_100_3 | 2.1 | 4.4 | 7.1 | 9.8 |

|  |  |  | Gradient Rate | | | |
| --- | --- | --- | --- | --- | --- | --- |
|  |  |  | 16% | 8% | 4% | 2% |
| Color | Shape | Identifier | Resolution | | | |
| Black | ● | 15_75_1 | 1.1 | 1.4 | 2.3 | 2.9 |
|  | ▲ | 15_75_2 | 1.0 | 1.4 | 2.2 | 2.9 |
|  | ■ | 15_75_3 | 1.1 | 1.3 | 2.2 | 2.7 |
| Blue | ● | 30_75_1 | 1.2 | 2.3 | 2.9 | 4.0 |
|  | ▲ | 30_75_2 | 1.1 | 2.1 | 2.8 | 3.6 |
|  | ■ | 30_75_3 | 1.2 | 2.4 | 3.4 | 4.6 |
| Red | ● | 60_75_1 | 1.7 | 3.2 | 4.5 | 5.0 |
|  | ▲ | 60_75_2 | 1.6 | 3.4 | 4.5 | 5.0 |
|  | ■ | 60_75_3 | 2.2 | 3.7 | 5.2 | 5.4 |
| Light blue | ● | 30_100_1 | 1.4 | 2.5 | 3.5 | 4.1 |
|  | ▲ | 30_100_2 | 1.5 | 2.5 | 3.6 | 3.9 |
|  | ■ | 30_100_3 | 1.4 | 2.3 | 3.2 | 3.7 |

**Table S4.** Resolution of PC (*Z* 18:1/18:1) and PC (18:0/18:2) at gradient rates 16%, 8%, 4%, and 2%.

**Table S5.** Resolution of PC (18:0/18:2) and PC (*E* 18:1/18:1) at gradient rates 16%, 8%, 4%, and 2%.

|  |  |  | Gradient Rate | | | |
| --- | --- | --- | --- | --- | --- | --- |
|  |  |  | 16% | 8% | 4% | 2% |
| Color | Shape | Identifier | Resolution | | | |
| Black | ● | 15_75_1 | 1.4 | 2.1 | 3.4 | 4.5 |
|  | ▲ | 15_75_2 | 1.3 | 2.0 | 3.3 | 4.6 |
|  | ■ | 15_75_3 | 1.4 | 1.9 | 3.4 | 4.2 |
| Blue | ● | 30_75_1 | 1.5 | 3.6 | 5.6 | 9.2 |
|  | ▲ | 30_75_2 | 1.5 | 3.4 | 6.5 | 8.8 |
|  | ■ | 30_75_3 | 1.5 | 3.2 | 5.1 | 7.3 |
| Red | ● | 60_75_1 | 1.8 | 4.1 | 6.3 | 7.6 |
|  | ▲ | 60_75_2 | 1.8 | 4.2 | 6.3 | 7.6 |
|  | ■ | 60_75_3 | 2.5 | 4.8 | 7.1 | 8.0 |
| Light blue | ● | 30_100_1 | 1.9 | 3.6 | 5.4 | 6.7 |
|  | ▲ | 30_100_2 | 2.0 | 3.6 | 5.4 | 6.4 |
|  | ■ | 30_100_3 | 1.8 | 3.3 | 5.0 | 6.0 |

**Table S6.** Resolution of PC (*Z* 18:1/18:1) and PC (*E* 18:1/18:1) at gradient rates 16%, 8%, 4%, and 2%.

|  |  |  | Gradient Rate | | | |
| --- | --- | --- | --- | --- | --- | --- |
|  |  |  | 16% | 8% | 4% | 2% |
| Color | Shape | Identifier | Resolution | | | |
| Black | ● | 15_75_1 | 2.5 | 3.4 | 5.9 | 7.4 |
|  | ▲ | 15_75_2 | 2.4 | 3.5 | 5.5 | 7.7 |
|  | ■ | 15_75_3 | 2.6 | 3.4 | 5.6 | 6.9 |
| Blue | ● | 30_75_1 | 3.0 | 5.8 | 7.7 | 10.4 |
|  | ▲ | 30_75_2 | 2.6 | 5.1 | 7.1 | 9.7 |
|  | ■ | 30_75_3 | 2.8 | 5.8 | 8.8 | 11.3 |
| Red | ● | 60_75_1 | 3.6 | 7.4 | 11.2 | 12.8 |
|  | ▲ | 60_75_2 | 3.5 | 7.5 | 10.1 | 13.3 |
|  | ■ | 60_75_3 | 4.7 | 8.7 | 12.7 | 13.6 |
| Light blue | ● | 30_100_1 | 3.4 | 6.2 | 8.9 | 10.9 |
|  | ▲ | 30_100_2 | 3.6 | 6.2 | 9.1 | 10.8 |
|  | ■ | 30_100_3 | 3.3 | 5.8 | 8.4 | 9.9 |

**Table S7.** Resolution of TG (18:1/18:1/18:1) and TG (18:0/18:0/18:0) at gradient rates 16%, 8%, 4%, and 2%.

|  |  |  | Gradient Rate | | | | |
| --- | --- | --- | --- | --- | --- | --- | --- |
|  |  |  | 16% | 8% | 4% | 2% |  |
| Color | Shape | Identifier | Resolution | | | | |
| Black | ● | 15_75_1 | 11.9 | 11.1 | 20.1 | 27.3 | |
|  | ▲ | 15_75_2 | 10.9 | 13.1 | 19.6 | 28.4 | |
|  | ■ | 15_75_3 | 10.3 | 12.8 | 19.9 | 26.5 | |
| Blue | ● | 30_75_1 | 12.0 | 18.7 | 24.1 | 36.7 | |
|  | ▲ | 30_75_2 | 10.1 | 15.8 | 26.5 | 35.9 | |
|  | ■ | 30_75_3 | 7.4 | 16.8 | 24.8 | 33.7 | |
| Red | ● | 60_75_1 | 12.6 | 20.7 | 32.0 | 42.8 | |
|  | ▲ | 60_75_2 | 11.8 | 19.2 | 32.0 | 42.8 | |
|  | ■ | 60_75_3 | 13.4 | 19.1 | 39.3 | 49.5 | |
| Light blue | ● | 30_100_1 | 11.4 | 18.2 | 32.6 | 38.0 | |
|  | ▲ | 30_100_2 | 11.0 | 16.2 | 29.1 | 40.9 | |
|  | ■ | 30_100_3 | 11.7 | 15.6 | 26.8 | 37.9 | |


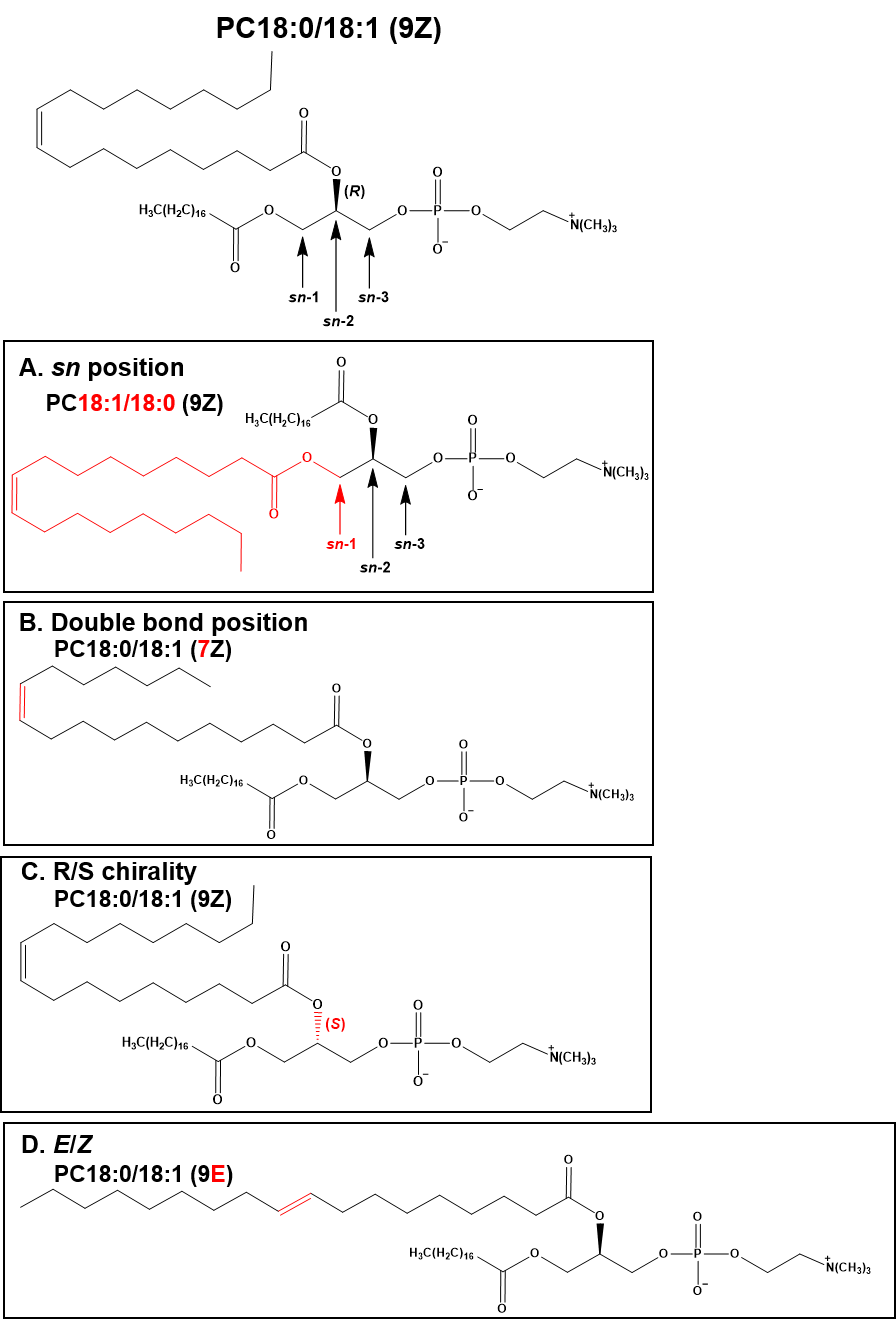


**Figure S1.** Example lipid PC18:0/18:1 (9*Z*) and its different types of isomers. Changes are

highlighted in red. A) *sn* isomerism entails switching the *sn* positions of the FA chains. B) double bond isomerism involves moving the double bond to a different position along the FA chain. C) *R*/*S* isomerism switches the chirality of the chiral carbon. D) *E*/*Z* isomerism changes the geometry of the double bond on the FA chain. The challenge of isomerism becomes even more complex when the FA chains contain multiple double bonds. In addition, isomerism can stem from FA chains that add up to the same number of carbons.

**Figure S2.** Selected reduced van Deemter curves of columns 15_75_2, 30_75_3, 60_75_3, and 30_100_1.**
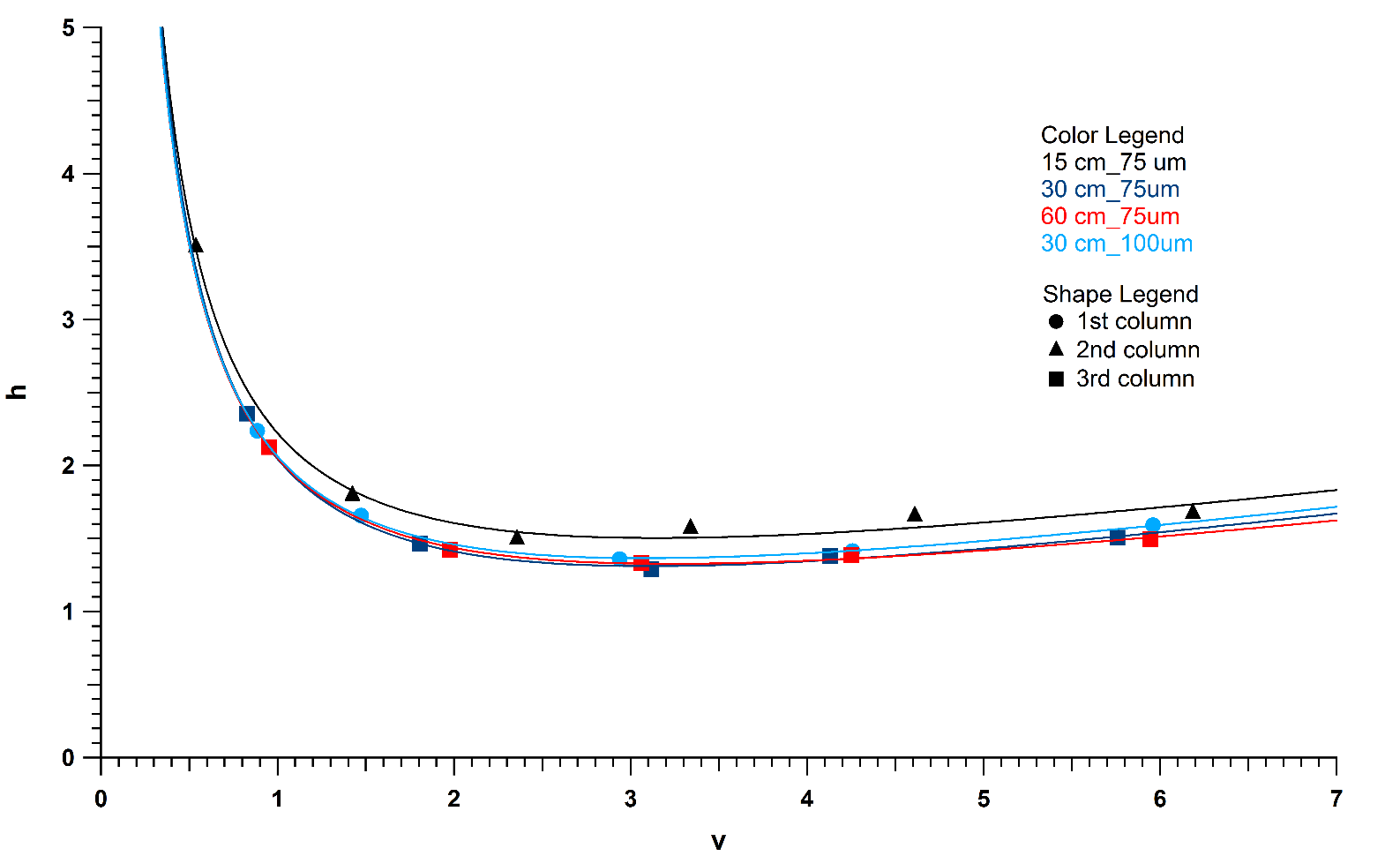
** The van Deemter curves were determined by test analyte hydroquinone (HQ) and the best fit of the van Deemter equation. Capacity factor (k’) of HQ is 0.2.


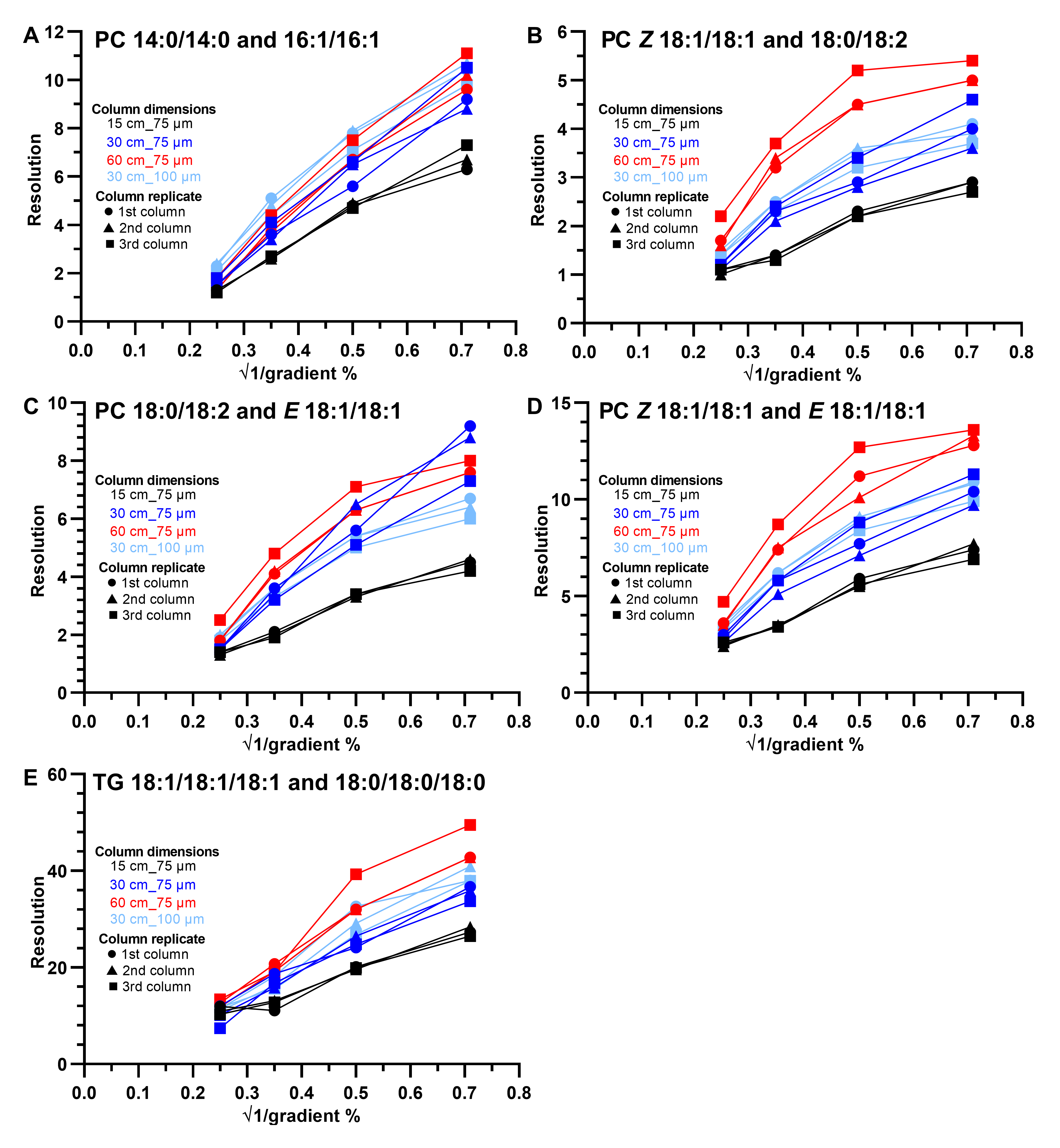


**Figure S3.** Resolution of each lipid pair is plotted against the square root of the reciprocal of each gradient rate. Three column replicates (circle, triangle, square) were analyzed for each set of columns (black, blue, red, light blue). A) PC (14:0/14:0) and (16:1/16:1). B) PC (*Z* 18:1/18:1) and (18:0/18:2). C) PC (18:0/18:2) and (*E* 18:1/18:1). D) PC (*Z* 18:1/18:1) and (*E* 18:1/18:1). E) TG (18:1/18:1/18:1) and PC (18:0/18:0/18:0).

## REFERENCES

[1] Hinz, H.-J., Thermodynamic Data for Biochemistry and Biotechnology. Springer-Verlag 1986.

[2] Tanaka, Y., Matsuda, Y., Fujiwara, H., Kubota, H., Makita, T., Viscosity of (water + alcohol) mixtures under high pressure. *Int. J. Thermophys.* 1987, *8*, 147–163.

[3] Godinho, J. M., Packing and characterization of capillary columns for ultrahigh pressure liquid chromatography, The University of North at Carolina Chapel Hill, 2016.

[4] Reising, A. E., Godinho, J. M., Jorgenson, J. W., Tallarek, U., Bed morphological features associated with an optimal slurry concentration for reproducible preparation of efficient capillary ultrahigh pressure liquid chromatography columns. *J. Chromatogr. A* 2017, *1504*, 71–82.
